# Targeting the Human 80S Ribosome in Cancer: From Structure to Function and Drug Design for Innovative Adjuvant Therapeutic Strategies

**DOI:** 10.3390/cells9030629

**Published:** 2020-03-05

**Authors:** Arnaud Gilles, Léo Frechin, Kundhavai Natchiar, Giulia Biondani, Ottilie von Loeffelholz, Samuel Holvec, Julie-Lisa Malaval, Jean-Yves Winum, Bruno P. Klaholz, Jean-François Peyron

**Affiliations:** 1 IBMM, Univ Montpellier, CNRS, IBMM, ENSCM, 34296 Montpellier, France; 2 Université de Strasbourg, CNRS, Inserm, Centre for Integrative Biology, IGBMC, 67404 Illkirch, France; 3 Université Côte d’Azur, Inserm, C3M, 06204 Nice, France

**Keywords:** ribosome, cancer, leukemia, antibiotics, targeted therapies

## Abstract

The human 80S ribosome is the cellular nucleoprotein nanomachine in charge of protein synthesis that is profoundly affected during cancer transformation by oncogenic proteins and provides cancerous proliferating cells with proteins and therefore biomass. Indeed, cancer is associated with an increase in ribosome biogenesis and mutations in several ribosomal proteins genes are found in ribosomopathies, which are congenital diseases that display an elevated risk of cancer. Ribosomes and their biogenesis therefore represent attractive anti-cancer targets and several strategies are being developed to identify efficient and specific drugs. Homoharringtonine (HHT) is the only direct ribosome inhibitor currently used in clinics for cancer treatments, although many classical chemotherapeutic drugs also appear to impact on protein synthesis. Here we review the role of the human ribosome as a medical target in cancer, and how functional and structural analysis combined with chemical synthesis of new inhibitors can synergize. The possible existence of oncoribosomes is also discussed. The emerging idea is that targeting the human ribosome could not only allow the interference with cancer cell addiction towards protein synthesis and possibly induce their death but may also be highly valuable to decrease the levels of oncogenic proteins that display a high turnover rate (MYC, MCL1). Cryo-electron microscopy (cryo-EM) is an advanced method that allows the visualization of human ribosome complexes with factors and bound inhibitors to improve our understanding of their functioning mechanisms mode. Cryo-EM structures could greatly assist the foundation phase of a novel drug-design strategy. One goal would be to identify new specific and active molecules targeting the ribosome in cancer such as derivatives of cycloheximide, a well-known ribosome inhibitor.

## 1. Introduction

In normal cells, protein synthesis (PS) is tightly linked to their proliferative needs [1,2]. In contrast, cancer cells have enslaved protein synthesis mechanisms to fuel their metabolic needs and a typical cancer cell expresses at least 10,000 different proteins [3], with PS being one of the most complex and energy-expensive cellular processes. All steps of PS are susceptible to dysregulation during cancer development, as previously reviewed [4,5]. Firstly, oncogenic signaling by mutated receptor tyrosine kinases (e.g., EGFR) and oncogenes (e.g., MYC, RAS) can converge at mTORC1 to stimulate the initiation of PS. Thereafter, initiation and elongation, two important molecular steps of PS, can occur at increased levels and this is achieved by the dysregulated expression of important translation factors [6]. For instance, the eukaryotic initiation factor 4F complex (eIF4F), which is critical to stimulate the initiation of PS, controlled by growth factors, is deregulated in cancer cells.

**Figure 1:**
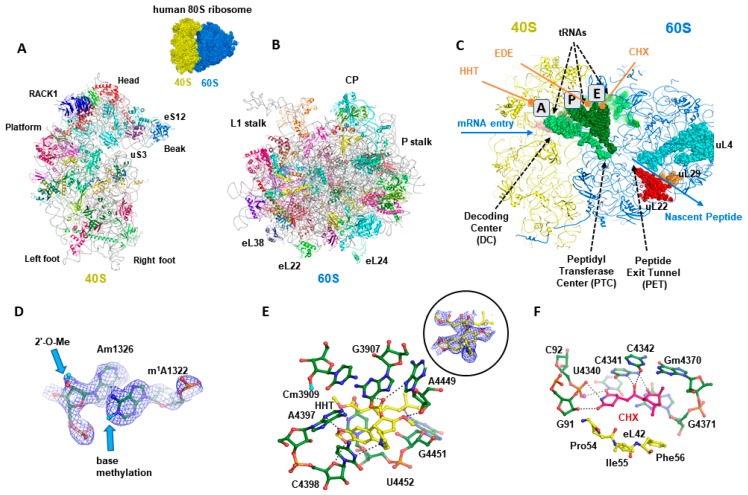
Structural analysis of the human 80S cytosolic ribosome. (**A**,**B**) Structure overview of the 40S and 60S ribosomal subunits of the human ribosome (atomic model derived from the cryo-EM structure of the 80S human ribosome [9,10]. (**C**) Typical functional sites on the ribosome, which are directly relevant for binding of inhibitors. (**D**) Detailed features in the high-resolution cryo-EM of the human 80S ribosome in which chemical modifications are visible [10]. (**E**) Structure of the human 80S ribosome complex with the HHT inhibitor (the inset shows the cryo-EM mpa density corresponding to the ligand). (**F**) Structure-based drug design: CHX as a case study; hydrogen bonds are indicated by dotted lines; eL42 is eukaryote-specific ribosomal protein which could be targeted to generate specificity using chemically modified ligands. CP: central protuberance; A: aminoacyl site; P: peptidyl site; E: exit site. CHX: cycloheximide; HHT: homoharringtonine; EDE: edeine.

The eukaryotic ribosome is the essential cellular nucleoprotein nanomachine of 3.5 to 4.5 Mega-Daltons [7] (Figure 1A,B) that decodes the genetic information carried by mRNAs into proteins, establishing a link between genes and cellular functions. In many, not to say all cancers, ribosome biogenesis is enhanced to face the important need for proteins by proliferating cancer cells [8]. As it is the central element of protein synthesis, it has been considered for a long time as a possible target for anti-cancer molecules. This review specifically focuses on the renewed interest in targeting the cytosolic human 80S (Svedberg constant) ribosome in cancer.

## 2. The Human Ribosome

In the mid-1950s, the cell biologist George Palade (1974 Nobel prize) identified granules at the surface of the endoplasmic reticulum membrane as the sites of protein synthesis in cells [11], that were named ribosomes in 1958 [12]. At that time, the first theory emerged: one gene-one ribosome-one protein, until a couple of years later it was realized that ribosomes were non-specialized structures which synthetize proteins from a mRNA template. It then took several decades to understand that ribosomes are highly complex structures and can exist as heterogeneous and specialized entities [13]. It also became clear that defects in ribosome function, regulation or composition, and more importantly, defects affecting protein synthesis, were involved in several human pathologies such as immunodeficiencies, metabolic disorders, neurological diseases, and particularly cancer (see [14] for an exhaustive review).

A plethora of structural studies on ribosomes with small ligand-bound molecules has provided a rationale for mechanistic inhibition of translation. Translation inhibitors specifically target catalytic sites or regulatory sites in the ribosome, to selectively interfere with one or more translation steps to eventually inhibit protein synthesis [7,15,16] (Figure 1C). Some antibiotics preferentially target either bacteria or eukaryotes or higher eukaryotes and some have precise selection towards cytoplasmic or mitochondrial ribosomes. Ribosomes display a conserved architecture that supports their function throughout the three kingdoms of life [17,18]. Similar to other ribosomes, the human ribosome comprises two subunits, a large (60S) and a small (40S) subunit, but human ribosomes are much larger than bacterial ones (4.3 MDa versus 2.3 MDa). The large subunit is composed of 3 rRNA chains (5S, 5.8S and 28S rRNA) and 47 ribosomal proteins, while the small subunit is composed of a single 18S rRNA chain and 33 ribosomal proteins, summing up to 80 different ribosomal proteins. Both subunits possess several functional sites, where various catalytic and regulation activities occur during protein synthesis. Initial translational activities are mediated by the small subunit, which carries the decoding center (DC), where codon-anticodon interactions take place. The large subunit mediates the catalytic translational activity, which occurs at the peptidyl transferase centre (PTC) that assembles amino acids (AA) into nascent polypeptidic chains according to the coding sequence in the mRNA. Further along, starting at the PTC, the nascent peptide chain progresses through the peptide tunnel to emerge from the ribosome. The ribosome possesses three tRNAs binding sites, the aminoacyl (A) site that binds the AA-charged transfer-RNA (tRNA), the peptidyl (P) site that is organized by the ribozyme activity of the 28S rRNA, and the exit (E) site with deacetylated tRNA (Figure 1C). All three tRNAs interact with both ribosomal subunits. The 3′-terminal CCA end interacts with the large subunit, whilst the anticodon stem of the tRNAs interacts with the small subunit to form base pairing interactions with the mRNA. In addition, both subunits possess various factor binding pockets, where the translation regulation activities occur, e.g., catalytic and regulation sites, inter-subunit bridges, which play a key role in ribosome structure and function. One of the roles of the ribosomal factors and proteins (including ribosomal GTPases) is to communicate between the two ribosomal subunits and to adapt and restrain their structural rearrangement during the translation process [9,18,19,20].

The human ribosome appears to be the most advanced structure in the ribosome kingdom, compared to ribosomes from lower eukaryotes or from bacteria [21]. Besides a core carrying essential functional sites, conserved throughout evolution, the human 80S ribosome possesses additional human-specific rRNA expansions segments (ES) and additional proteins. In particular, the identity and function of “internal“ RPs associated with the core structural and enzymatic functions do not vary between ribosomes from different species, in contrast to RP localized on the surface [22], raising the possibility of new environmental functions of the 80S ribosome. Evidence exists that specific ES and RPs could be involved in the selection of specific mRNAs for translation, in interactions with different upstream regulators and in defining specific cellular localizations [13,21]. The identification of hundreds of ribosome-associated proteins (RAPs) defined as a “ribo-interactome“ further supports diverse interactions of the ribosome with its cellular microenvironment, likely influencing its functions and the quality/specificity of translation [23].

In addition, the ribosome is subjected to many post-transcriptional and translational chemical modifications such as ribose 2′-OH methylation, pseudouridination (Ψ) (Figure 1D) and several other base modifications of rRNAs. Chemical modifications that occur as soon as during ribosome biogenesis alter the properties of RPs and rRNAs and likely participate in modulating ribosome heterogeneity and function in the cells [13]. The human ribosome evolved to consist of more than 200 chemical modifications which are introduced in its rRNAs during ribosome biogenesis [10,24,25]. Evidence exists that occurrence and sub-stoichiometry of chemical modifications vary according to environment, tissue types, cell development, diseases and pathological conditions [10,13,25,26,27,28,29]. In addition, alteration in sets of 2′-O-Me sites and Ψs stoichiometric level has a greater impact on ribosome function than ribosomes with a single site sub-stoichiometry [30,31,32,33].

## 3. The Ribosome in Cancer

### 3.1. Ribosomal Genes Mutations in Cancer

Genetic defects that lead to defective ribosome functioning or altered production of ribosomes can generate ribosomopathies. This was among the first observations linking ribosomes to cancer, as most of these congenital diseases are associated with a higher risk of developing cancer. The defects can concern ribosome biogenesis (Shwachman-Diamond syndrome, Dyskeratosis congenita) or directly affect RPs (Diamond-Blackfan Anemia), see a recent comprehensive review [34].

Ribosomopathies/mutations in RP genes mostly affect the bone marrow tissue containing highly proliferative hematopoietic cells and are associated with a reduced number of ribosomes or to a non-optimal ribosomal function. These defects first produce a hypoproliferation state that paradoxically ends up with time into a hyperproliferation state that supports a malignant transformation. First, the impaired ribosome function decreases cell proliferation and generates a ribosomal stress that converges to a p53 salvage response to eliminate ribosome-deficient cells through apoptosis. However, because these cells have a high proliferative potential and despite the fact that they carry malfunctioning ribosomes, some could accumulate compensatory genetic defects that produce a cellular advantage and become drivers of transformation. Alternatively or concomitantly, the defective ribosomes could express a different mRNA “translatome”, favoring the translation of cancer-promoting mRNAs for proteins or proto-oncogenes and therefore pushing the cells into transformation [28,35].

This nascent ribosome-cancer connection was further consolidated by the observation of several somatic mutations affecting RP genes in various cancers such as leukemia, gastric, brain and ovarian cancers [36]. Mutations in RP genes uL18 (old nomenclature: RPL5); uL16 (RPL10); uL5 (RPL11) and eL22 (RPL22) have been observed in 20% of T-ALL, see [37] for a review. Proteins uL18 and uL5 have demonstrated extra-ribosomal functions to modulate the p53 pathway [38]. Moreover, the R98S mutation in uL16/RPL10 results in a dysregulation of the JAK-STAT signaling pathway [39].

### 3.2. The Ribosome as an Important Actor in Cancer

The research to define the role of ribosomes during malignant transformation evolved from the question asked in 2003 by Ruggero and Pandolfi “Does the ribosome translate cancer?“ [40] to the 2017 review of Sulima et al.: “How ribosomes translate cancer“ that highlighted how the ribosome can now be considered not only as an important player in cancer development [41] but also as a potential target as also demonstrated by the anti-leukemic effects displayed by eukaryotic-specific antibiotics [42].

It has been known for decades that morphological changes of the nucleolus, the nuclear compartment where rRNAs are transcribed and associate with RPs to form ribosomal subunits, are markers of cancer transformation [43], used by pathologists to support their diagnosis [44]. These changes at the nucleolus level are signs of an intense ribosome biogenesis that is augmented in cancer cells by many mechanisms [8,34]. The first event in ribosome biogenesis is the transcription of rRNA in the nucleolus by RNA polymerase I (Pol I) whose activity varies during the cell cycle [45]. UBF (Upstream Binding Factor) is an important basal factor required for efficient transcription of ribosomal genes, that appears to funnel various stimuli intended to enhance Pol I activity and ribosome biogenesis. It is activated via multiple phosphorylation events by different kinases such as casein kinase II (CKII) which is overexpressed in many cancers. Additionally, UBF integrates cytosolic clues after being phosphorylated by Erk1/2 and is coordinated to the cell cycle by the Cdk4-CycD1 and Cdk2-CycE complexes, which can also be upregulated in cancers. On the other side of the balance, the tumor suppressor phosphatase PP2A represses phosphorylation of UBF to restrain ribosome biogenesis and cell growth.

Defects in two other tumor suppressors, p53 and Rb, lead to the aberrant upregulation of Pol I and Pol III activity to stimulate ribosome biogenesis and support tumor growth and development [40]. Chronic inflammation has also been proposed to favor the early steps of tumor initiation [46,47]. Interestingly, a link between inflammation and ribosome biogenesis has been discovered, through the inflammatory IL6 cytokine that stimulates, in a c-MYC-dependent manner, transcription of rRNAs. These rRNAs will then aggregate with RPs to form new ribosomes, decreasing therefore the number of RPs that can interact with and block MDM2, preventing the degradation of p53 [48].

Not only protein synthesis rate is correlated with tRNA abundance, but also the composition of the tRNA pools expressed by a cell and their usage by ribosomes, can influence the translated proteome and, as a consequence, the cell’s fate. For instance, two different translation programs have been associated with either proliferation or differentiation [49]. Coordinated changes were observed between mRNAs and tRNAs, to adapt the signature codon usage of a tRNA pool to the signature codon usage of the genes that are expressed in a particular cellular state, linking in this way the translational code to the genetic code. Changes in mRNA between proliferation/differentiation were mirrored by changes in tRNA supply. tRNAs expressed in proliferative cells carry anti-codons that correspond to the codons present in proliferation-associated genes and were repressed in differentiated cells. In the reverse situation, mRNA from differentiated cells match a pool of tRNA with corresponding anti-codons, that are up-regulated in differentiated cells but down-regulated in proliferative ones to ensure a high and efficient translation of the mRNA that are important for the particular cellular state. Through high-throughput tRNA profiling in breast cancer, an enrichment in specific tRNAs was observed, that favors the expression of a pro-metastatic mRNA program, to promote metastatic progression [50].

**Figure 2:**
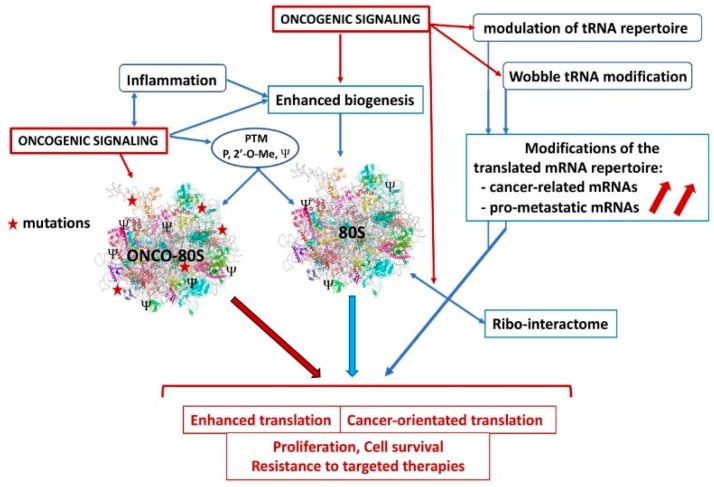
Dysregulations affecting ribosome functions in cancer. Oncogenic signaling and chronic inflammation can stimulate ribosome biogenesis and/or alter post-translational modifications, to stimulate translation efficiency or specificity. The supposed 80S oncoribosome could bear mutations, display differential expression of some critical RPs for enhanced activity or difference in the translated mRNA repertoire, favoring cancer cell metabolism. Oncogenic signaling could modulate the tRNA repertoire and the translation selectivity (Wobble effect) to favor expression of cancer and pro-metastatic mRNAs. Modifications of the ribo-interactome by oncogenic influences could favor the translation of a cancer-related mRNA repertoire. PTM: post-translational modification, P: phosphorylation, 2′-O-Me: methylation, Ψ: pseudouridination.

Wobble tRNA modifications involving the U_34_ enzymes that are upregulated by the *BRAF^V600E^* oncogene in melanoma were shown to participate in resistance to targeted therapies. Wobble U_34_ modifications are required for the ribosome to decode AAA, GAA and CAA codons that are significantly enriched for the HIF-1A transcription factor. By acting with U_34_ enzymes, *BRAF^V600E^* was found to reprogram melanoma cells towards an HIF1A-dependent shift to glycolysis and hypoxia, generating resistance to therapies [51]. Figure 2 shows the dysregulations affecting the ribosome in cancer cells.

### 3.3. Targeting Ribosome Biogenesis

Ribosome biogenesis represents a control checkpoint for progression in the cell cycle. The *MYC* protooncogene acts as a major inducer of protein synthesis in cancer cells by sustaining ribosome biogenesis through the stimulation of DNA PoI II and III activity and through increase in the levels of translation factors, and ribosomal DNA [52,53]. A lack of proper ribosome biogenesis is associated with the production of free RPs, a prototype being ribosomal protein uL5, that then interacts with HDM2/MDM2, interfering with its function to degrade p53, preventing a tumor-suppressive response [54,55]. Ribosome biogenesis is therefore considered as an attractive anti-cancer target [56]. As a matter of fact, it has been realized that interference with ribosome biogenesis was part of the anti-cancer properties of several classic anti-neoplastic drugs, even if the ribosome was not considered as their primary target [57]. Several of these antibiotic drugs, called anti-neoplastic antibiotics, come from the anthracycline family, widely used against cancer. They act as DNA intercalating agents to interfere with several steps of rRNA synthesis. Besides, for drugs of the platinium family, it has been recently shown that oxaliplatin affects cancer cells by triggering a ribosome biogenesis stress response that leads to cell death, in contrast to cisplatin and carboplatin, that instead act through an induction of DNA damage; likely explaining the differences in tumor type selectivity and side-effect profiles [58].

These observations have been the basis for the development of specific inhibitors of Pol I to interfere with transcription of ribosomal genes. Inhibitors such as CX-5461 or BMH-1 have shown interesting anti-cancer activities in various cancer models through two different modes of action, CX-5461 by interfering with rDNA quadruplexes and BMH-1 by binding to GC-rich sequences enriched in ribosomal genes [34].

### 3.4. Ribosomes: Multifaceted Targets

As a proof of principle to validate ribosomes as potential anti-cancer targets, it has been shown that cancer cell proliferation may be inhibited by conditional deletion of the *eS6 (S6RP)* gene, that globally decreased ribosome function whilst nutrient sensing and growth were unaffected [59]. Moreover, the mandatory role of the ribosome in *Myc*-dependent transformation was elegantly demonstrated as haploinsufficiency of the eL24 (RPL24) or eL38 (RPL38) ribosomal proteins prevented lymphoma induction in a transgenic in EµMyc mouse model [60]. Interestingly, this tumor-suppressive effect required the functional p53 pathway. These genetic approaches elegantly suggest that pharmacological interference with the ribosome function may potentially be a valuable anti-cancer strategy.

The human 80S ribosome is structurally complex, harboring binding sites/pockets for mRNA, tRNA and ribosomal factors whose normal mechanism of action can be modified by small inhibitory ligands to interfere with ribosome function. Translation and protein synthesis involve precise and complex processes that proceed at high speed but, nevertheless, with high fidelity through the four stages of initiation, elongation, termination and recycling [61]. It turns out that the ribosome is one of the main targets of natural or synthetic antibiotics. These act by binding at the various functional centers to lock the ribosome in a particular conformation, thereby preventing the access of tRNAs or by interfering with the actions of translation factors [62]. The progress in structural biology has identified at the atomic level how some antibiotics are binding to eukaryote ribosomes from either yeast [16] or humans [42]. This knowledge could become useful in developing new ribosome ligands as novel therapeutics for infectious diseases (using prokaryote-specific antibiotics), genetic disorders and particularly for new cancer treatments (using eukaryote-specific antibiotics and inhibitors, see below).

Targeting protein factors could also be an attractive option. For example, the eIF5A elongation factor is post-translationally modified by the addition of the unusual amino acid hypusine [63]. This modification is crucial for the ribosome to synthesize proteins with poly-proline stretches. Interfering with hypusination of eIF5A with GC7, an inhibitor of deoxyhypusine synthase (DHPS), could be a potential approach that could affect cancer cells with a high rate of protein synthesis. Additionally, it could be a way to interfere with ATG3 translation which requires hypusination, in order to prevent autophagy and to interfere with cancer cells survival [64].

### 3.5. Who Is the Best Target in Cancer: 80S Ribosomes or Mitoribosomes?

**Figure 3:**
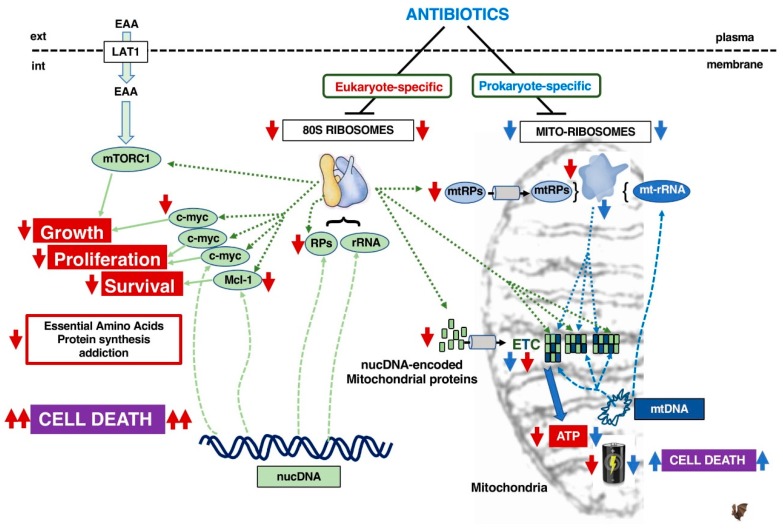
Consequences of targeting 80S ribosomes vs. mitoribosomes with respectively eukaryote-specific antibiotics (red arrow) or prokaryote-specific antibiotics (blue arrow). The style of the connecting lines defines the type of function involved: translation by ribosomes (........), transcription (-------), biological effect (^_____^). EAA: Essential Amino Acids, LAT1: System L amino-acid transporter 1, RPs: ribosomal proteins, mtRPs: mitochondrial ribosomal proteins, nucDNA: nuclear genome DNA, mtDNA: mitochondrial DNA, rRNA: ribosomal RNA, mt-rRNA: mitochondrial ribosomal RNA, nucDNA-eMP: nuclear DNA-encoded mitochondrial proteins, ETC: electron transport chain.

Eukaryotic cells express two types of ribosomes, the cytosolic 80S as well as the 55S mitoribosome in mitochondria. While 80S ribosomes translate all cellular proteins, mitoribosomes exclusively translate some mitochondrial components of the electron-transport chain (ETC) that produces cellular energy and ATP. For example, 13 proteins of the electron transport chain are encoded by the mtDNA and are translated by mitoribosomes whilst the remaining 70 are encoded in the nucleus and translated by 80S ribosomes. Mitoribosomes are composed of mt-rRNAs that have a mtDNA origin, whereas mt-RPs are encoded in the nucleus and translated by 80S ribosomes (Figure 3). 

Since mitochondria have a bacterial origin, mitoribosomes are supposed to be evolutionarily related to bacterial ribosomes and it is therefore proposed that the use of prokaryotic (P)-antibiotics affecting protein synthesis in bacteria will interfere with mitochondrial function, to damage oxidative phosphorylation (OXPHOS). Consequently, this may kill cancer cells that frequently rely on OXPHOS for energy production [65]. Moreover, several recent studies show that various P-antibiotics (used at a rather high concentration of 100 µM) could affect in vitro proliferation, survival and the cancer stem cell properties of various immortalized cell lines, representing diverse human cancers (breast, ovary, colon, prostate, lung, pancreas, as well as melanoma and glioblastoma) [66,67]. In addition, it has been reported that the bacteriostatic agent tigecycline has an anti-cancer activity which is due to its ability to inhibit mitochondrial translation in leukemia [68], ovarian cancer cells [69], renal cancer [70] and imatinib-resistant CML cells [71]. However, AML cells have been shown to escape the tigecycline-induced OXPHOS damage by rapidly and reversibly shifting their metabolism and ATP production towards glycolysis [72]. This suggests that targeting ETC in cancer cells by inhibiting mitoribosomes might not be effective to eradicate metabolically plastic cancer cells.

Moreover, mitoribosomes may not be as similar to bacterial ribosomes as first imagined. Mitochondria appear to have evolved from multiple endosymbiotic events between an α-proteobacteria and a primordial eukaryote. This symbiosis was associated with mutual gene transfer from the mtDNA to the host genome, with the acquisition by the mitochondria of host proteins [73]. Mitoribosomes appear structurally very different from bacterial ribosomes as revealed by cryo-EM experimental approaches [74,75]. Compared to bacterial ribosomes, they have a higher r-protein to rRNA ratio as their rRNA has been reduced by 40% due to various deletions that greatly affect the secondary structure. Furthermore, the 5S rRNA found in all “standard“ prokaryotic and eukaryotic ribosomes is absent. As a consequence, several RPs are also absent because of the lack of their respective rRNA binding sites. Nevertheless, the overall mass of 55S is conserved depicted by a small subunit of 28S and a large one of 39S, due to the presence of new mitochondrial-specific polypeptides that stabilize and change the overall structure [76]. Although the active functional centers of the ribosomes are highly conserved within the species, structural differences may affect the binding of prokaryote-specific antibiotics to mitoribosomes.

In leukemia, 80S ribosomes occur at the crossroads of at least two important transformation pathways: the mTORC1 which acts downstream of the PTEN-PI3K-Akt axis to stimulate translation initiation, and the c-MYC proto-oncogene, frequently amplified in cancer/leukemia, which acts by stimulating many molecular steps of PS and increasing the number of ribosomes (see below).

Importantly, we observed that eukaryote-specific (E)-antibiotics within the 10µM range had strong anti-leukemic activity, which was not observed for P-antibiotics used at the same doses and time scale [42]. This higher efficacy might be explained by the E-antibiotics impacting both crucial short-lived proteins such as c-MYC and MCL-1 involved in stimulating cancer proliferation and survival, as well as mitoribosomes whose mt-r-proteins are translated by 80S ribosomes. The latter will indirectly target the components of the mitoribosome-translated ETC components.

We, therefore, are in favor of the idea that specific inhibitors of the 80S ribosome will affect a larger panel of cellular substrates, including mitoribosomes, components of the ETC and short-lived oncogenes when displaying potent anti-cancer effects.

## 4. Direct Targeting of the 80S Ribosome

Targeting the ribosome and consequently blocking protein synthesis aims to deleteriously affect against cancer cells but this may be also damaging for normal cells, particularly those in the proliferation phase. As cancer cells nevertheless frequently develop a strong addiction to protein synthesis, they are preferentially expected to have increased sensitivity to ribosome inhibition, compared to normal cells. This addiction is further supported by the constitutive activation of the mTORC1 pathway observed in many cancers. This signaling stimulates anabolic reactions and, in particular, protein synthesis and translation initiation [77]. There are many examples depicting a strong induction of cancer cell death triggered by starvation. For example, in T-cell acute leukemia, we demonstrated that blocking the uptake of essential amino acids that fuels constitutive mTORC1 activation and the stimulation of protein synthesis, induces a strong apoptotic response [78].

Moreover, many proteins with oncogenic potential such as c-MYC have a short half-life (15 min) and are particularly and rapidly affected by ribosome inhibition, compared to most proteins with a longer half-life. We postulate that ribosome inhibitors could be highly effective against cancer cells, which depend on such short half-life oncogenic proteins. There are already several examples in the literature to support this hypothesis (see below).

Similarly, we previously showed that targeting the 80S ribosome with eukaryote-specific antibiotics strongly decreased c-MYC levels resulting in cell death. Under the same conditions, normal lymphocytes were unaffected [42]. c-MYC is a strong oncogenic driver in many cancers including acute leukemia [79]. Decreasing its levels is likely to reduce proliferation and survival influences, supporting the interest to associate ribosome inhibitors to classical chemotherapies.

### 4.1. Homoharringtonine, a Clinically Used Ribosome Inhibitor

**Figure 4:**
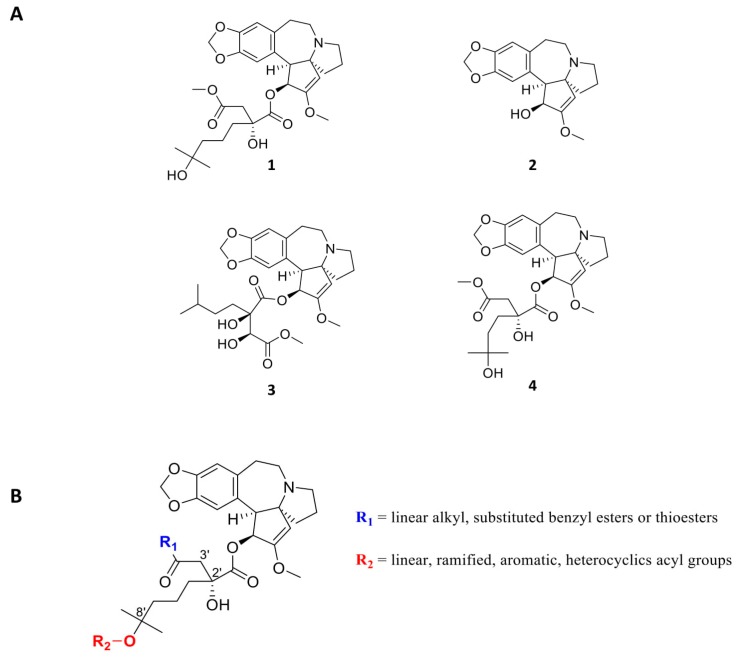
(**A**) Structures of Homoharringtonine (omacetaxine mepesuccinate, Synribo^®^) **1**, Cephalotaxine **2**, Isoharringtonine **3** and Harringtonine **4**. (**B**) Chemical modifications of Homoharringtonine.

Many antineoplastic drugs are derived from natural products: synthetic and medicinal chemistry therefore plays a central role in the process leading to marketed drugs [80]. Homoharringtonine (Figure 4A, compound 1) is a cephalotoxine ester that was discovered in 1963 from *Cephalotaxus harrigtonia*, including cephalotaxine (compound 2), isoharringtonine (compound 3) and harringtonine (compound 4) [81]. Homoharringtonine (called omacetaxine mepesuccinate in its semi-synthetic form) is hemi-synthesized from cephalotaxine by an esterification reaction, which is currently being investigated for improved synthesis [82,83].

Biochemical experiments testing the effect of HHT on the ability of human 80S from placenta to synthesize diphenylalanine in presence of poly-U mRNA, elongation factor eEF1 and Phe-tRNA^Phe^, first predicted HHT to interfere with the acceptor site of the tRNA [84]. Thereafter, the crystal structures of HHT bound to the archaeal ribosome showed that HHT binds to the A-site cleft and thus prevents the incoming A-site tRNA from accommodating its CCA end into the PTC [16,85]. Comparison of the *E. coli* and eukaryotic ribosome structures revealed a structurally more constrained pocket for HHT in the bacterial ribosome that prevents HHT binding. Further analysis showed that the translation inhibitory effect of HHT is much higher in eukaryotes than in archaea, further supporting HHT to be a eukaryote-specific inhibitor [85]. Additional structural analysis of HHT bound to the yeast ribosome and to the human ribosome essentially confirmed its binding site and mode of action, shown in Figure 1 [10,16].

HHT was shown to have anti-proliferative activity on murine leukemic cells [86] with the highest activity in vitro on leukemic cell lines. Following several trials in China, HHT was approved by the FDA in the U.S. in 2012 for the treatment of Chronic Myeloid Leukemia (CML) patients who experienced a resistance to at least two tyrosine kinase inhibitors (TKI). Its anti-cancer activity is currently actively being studied (20 articles in 2019, almost all of them concerning acute myeloid leukemia, PubMed source).

From a medicinal chemistry point of view, a tremendous amount of plant extraction analysis and synthetic work has been done during the past 40 years concerning cephalotaxine esters and HHT derivatives: almost 60 molecules have been extracted from natural sources, tested, and their structures resolved [87]. In their patent, Bataille et al. have exemplified and tested about 65 analogues [88]. More than 20 different ester groups in the 4′ position have been synthesized, and are retaining HHT activity. Acylated derivatives in the 8′ position also indicated very good bioactivity level. The other positions must remain untouched in order to avoid a critical drop in activity (Figure 4B). Many different synthetic strategies have been established in order to either increase the synthesis scale by straightforward synthesis or to ease the development of future synthetic analogues for drug development [89]. HHT is particularly interesting because of its history, as it is the first natural compound to bind directly to the ribosome, inhibit protein synthesis and used in cancer therapy. It is used as a reference in the research of new anti-cancer ribosome inhibitors. Most of these studies have been performed without an exact understanding of the HHT target. However, now that the 80S ribosome structure has been resolved, it is possible to manipulate the structure of HHT in order to increase its bioactivity, as well as its metabolic stability.

The anti-cancer properties of HHT that are described below represent valuable proofs of concept for the use of ribosome blockers to alter cancer growth and demonstrate that the inhibition of the ribosome per se can be tolerated in humans, at least during short term treatments. One can then wonder why HHT is the only ribosome inhibitor from many known compounds that successfully entered the clinic? All these molecules display a great variety of structures and of modes of action, acting at different sites on the ribosome [16], with different selectivity for different ribosomes (80S, bacterial ribosome, mitoribosome) that define their inhibitory potential. It is therefore possible that other inhibitors have a lower inhibitory activity than HHT, which can be associated with side effects outside the ribosome, thus generating toxicity.

### 4.2. Ribosome Inhibition in Hematopoietic Malignancies

HHT appears to be highly efficient in Chronic Myeloid Leukemia cells as it can act on several proteins involved in the transformation process. HHT strongly affects the levels of the BCR-ABL oncogenic tyrosine kinase because of its high turnover rate, and as a consequence potentiates the effect of the tyrosine kinase inhibitor (TKI) imatinib [90,91]. HHT also impacts on c-MYC and the anti-apoptotic Bcl-2 family member MCL-1. These two proteins have a short half-life and support proliferation and survival of CML cells [91]. HHT was shown to affect the cellular protein and mRNA levels of the EPHB4 tyrosine kinase. EPHB4 appears to be one element of the resistance of CML cells to imatinib in a cellular model that consists in two cell lines established from the diagnostic and relapse stages of a unique patient [92]. Interestingly, in 2012, HHT/omacetaxine was shown to be a safe and effective alternative treatment in a phase 2 study on 62 CML patients bearing the T315I BCR-ABL mutation [93], which is associated with resistance to all clinically available TKIs [94].

In Multiple Myeloma, the anti-tumoral effect of HHT occurs as a result of a significant reduction in the levels of MCL-1. HHT also reduced ß-catenin and XIAP levels to interfere with disease progression [95]. Although it was not formerly demonstrated that the downregulation of MCL-1 and ß-catenin by HHT relied on ribosome inhibition, the effects were still observed upon the blockading of apoptosis.

In Acute Myeloid Leukemia, mutations on the FLT3 tyrosine kinase receptor, such as FLT3-ITD are associated with a poor outcome in patients [96]. HHT emerged from a drug screening approach as a molecule with preferential anti-leukemic effects on FLT3-expressing AML cells and displayed a synergistic action with the multi-kinase inhibitor sorafenib (Sor). Interestingly, a phase 2 trial evaluating HHT+Sor in 24 patients with either relapse or refractory AML showed the achievement of a complete remission (CR)/complete remission with incomplete hematology recovery (CRi) in 20 patients. HHT strongly decreased the levels of short half-life FLT3-ITD, possibly by ribosome inhibition and diminished downstream FLT3 signaling events such as STAT5 phosphorylation, an event associated with stimulation of cell proliferation [97].

### 4.3. Ribosome Inhibition in Various Solid Cancers

Non-Small Cell Lung Carcinoma, NSCLC, is driven by an oncogenic version of the EGF receptor and can be treated with Gefitinib until relapse fatally occurs within 6–12 months. HHT displayed some anti-cancer activity in vitro on NSCLC cellular models including Gefitinib-resistant variants. The HHT mode of action in this cancer has not been precisely demonstrated although HHT decreased the cellular levels of MYC, MCL-1, SURVIVIN and also interfered with JAK1 and STAT3 phosphorylation, to decrease the levels of IL6, a growth factor for NSCLC [98].

In triple negative breast (TNBC) cancer cell lines, HHT was demonstrated to strongly decrease survival influenced by MCL-1, BCL-2, SURVIVIN, XIAP and could reduce cancer growth of MDA-MB-231 xenografts [99].

### 4.4. Ribosome Inhibitors and Metastasis

Invadosomes are cellular dynamic F-actin structures that are involved in cell adhesion, signaling and migration [100]. They are key elements in the metastatic invasion process of solid tumors. Their capacity to degrade the extracellular matrix in favor of cancer cell dissemination and their presence at the surface of cancer cells correlates with invasiveness. A laser microdissection approach combined with mass-spectrometry showed that invadosomes from fibroblasts are enriched for components of the protein translation machinery including initiation/elongation factors and RPs, thereby favoring the presence of delocalized ribosomes. This could be responsible for the translation of specific mRNAs coding for proteins involved in invadosome formation and favoring metastasis. Interestingly, treatment of cancer cells with the protein synthesis inhibitor anisomycin, or invalidation of translation-related proteins by siRNA, both destroyed invadosomes [101]. Targeting ribosomes in invadosomes could thus be a means to interfere with invasion and metastasis. Moreover, the identification of the key proteins specifically translated by these specific ribosomes could shed new light on the invasion process itself.

### 4.5. Ribosome Inhibitors and Immunotherapies

Stimulation of both the innate and the adaptive effector arms of the immune system has emerged as a powerful new therapy for cancer. One of the mechanisms developed by cancer cells to evade the immune system is through expression of inhibitory receptors that interfere with T cell activation. Two major pathways are currently being explored within this field: CTLA-4 (cytotoxic T lymphocyte-associated protein 4) and PD-1 (Programmed cell death 1). Several antibodies that block the interaction CTLA-4 with CD80/CD86 or of PD-1 with PDL-1 to unleash T cell activation, are being used in clinics; please see [102] for the review. Many patients, however, do not respond to checkpoint inhibitors, suggesting that additional or combination therapies could be further explored. Two interesting studies suggest that interfering with protein translation could improve immunotherapies [103].

It was shown that interfering with the function of the eIF4F complex resulted in a decreased expression of PDL-1 either after interfering with translation of STAT1 leading to a lower transcription of PDL-1, or via interfering with eIF4E phosphorylation and activity, in melanoma [104] or liver cancer [105]. These studies demonstrate that checkpoint inhibitors can be translationally modulated and that ribosome inhibitors could be relevant for such a role.

In a mouse lung tumor model driven by a KRAS mutant, HHT exhibited anti-tumoral effects by decreasing IL12 levels and promoting B-cell activation and anti-tumor activity, although the exact mechanisms remains to be further explored [106].

**Figure 5:**
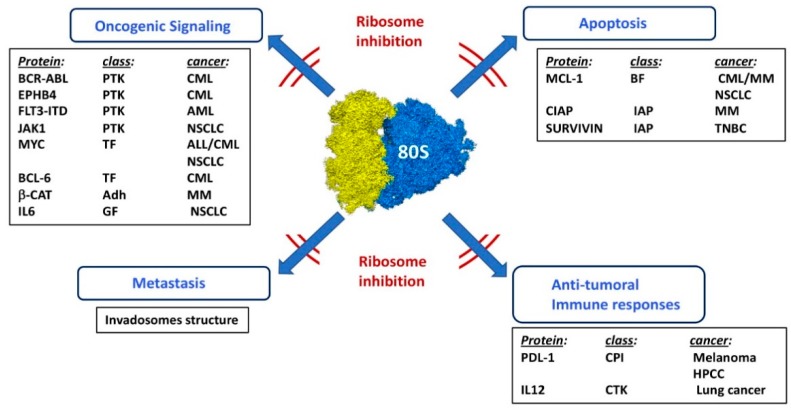
Effects of ribosome inhibition in various cancer models. Combination of ribosome inhibition with other chemotherapeutic treatments. PTK: protein tyrosine kinase, TF: transcription factor, Adh: adhesion molecule, GF: growth factor, BF: BCL-2 family, IAP: inhibitor of apoptosis, CPI: check point inhibitor, CTK: cytokine, CML: chronic myeloid leukemia, AML: acute myeloid leukemia, NSCLC: non-small cell lung carcinoma, MM: multiple myeloma, TNBC: triple negative breast cancer, HPCC: hepatocarcinoma.

Figure 5 illustrates the results presented in the above sections.

### 4.6. Combining Ribosome Inhibition with Classical Chemotherapeutic Drugs

HHT appears to potentiate the anti-cancer effects of many known drugs. In AML, HHT produced additive or synergistic effects when combined with the anti-metabolite AraC, or the anthracycline aclarubicin [107,108] and with the methylation inhibitor decitabine [109]. It synergized with abivertinib, a new inhibitor of BTK that interferes with signaling events (phosphorylation of Btk, Akt, IKK, Flt3, STAT5) in AML cellular models to amplify leukemic cell death [110].

HHT had synergistic effects with a Heat Shock Protein 90 inhibitor on FLT3-ITD+ AML models [111], with etoposide on primary and AML cell lines [112]. HHT synergized with oridonin on t(8; 21) AML cells by downregulating signaling via c-KIT and decreasing levels of the multi drug resistance proteins MRD1 and MRP1, in order to increase the effective concentration of oridonin [113]. HHT furthermore synergistically amplifies apoptosis when combined with SAHA, a histone deacetylase inhibitor by upregulating the expression of death receptors at the surface of AML cells [114]. The combination of HHT with arsenic trioxide was efficient to kill CD34^+^CD38^−^ cells in the KG-1 and Kasumi-1 AML cell lines and in primary cells, suggesting that the treatment could affect Leukemic Stem Cells that maintain the disease and are responsible for relapse. Although, this assumption would need more in vivo experiments [115].

HHT cooperates with the proteasome inhibitor bortezomib to kill Diffuse Large B Cell Lymphoma (DLBCL), particularly by targeting MCL-1 [116]. The effect of HHT on MCL-1 can be combined with targeting of Bcl-2 with ABT-199 to produce a marked synthetic lethality in BCL-2+ DLBCL samples [117]. These results are summarized in Table 1.

## 5. Advent of High-Resolution Cryo-EM Allows Structure-Guided Drug Design

Most of the antibiotics could be used as anti-cancer compounds, but their inherent toxicity strongly alters this possibility. They have been used in cancer therapy since the late 50s; for example, actinomycin D (also called Dactinomycin) was the first antibiotic used to treat some types of cancer [118], despite its high level of toxicity [119]. Nevertheless, almost all antibiotics, and more generally, antimicrobial agents, have been empirically used for cancer therapy, thus lacking rational design.

Cryo-electron microscopy (cryo-EM) has become a key method to determine the structure of biological macromolecules and complexes. In recent years, with the so-called “resolution revolution” [120] made possible by new highly sensitive direct electron detector cameras and better software for image processing, cryo-EM has become preferred for high-resolution structure determination. This was highlighted by the 2017 Nobel Prize for chemistry awarded to Jacques Dubochet, Joachim Frank and Richard Henderson. The two actual limitations of cryo-EM include the relatively low throughput of the technique, as well as the long time required to obtain a high-resolution structure, which are being resolved at the levels of sample preparation and on-the-fly data processing, in order to produce faster workflows. By overcoming such bottlenecks, high-resolution cryo-EM structures could be obtained faster, making cryo-EM a new tool for structure-guided drug design. We have shown that cryo-EM is a promising tool to analyze ligand complexes with the human ribosome [10,42]. Thus, in addition to avoiding a limiting crystallization step, it is now technically feasible to obtain structures by cryo-EM at a resolution where drug interactions can be analyzed. In addition, it opens the possibility to analyze a variety of functional complexes with mRNA and ribosomal factors and provide insights into the mechanism of inhibition of the human ribosome induced by ligands. Based on these structures, it becomes possible to perform computational analysis of the complexes to perform structure-guided drug design.

In 2020, more than 10,000 cryo-EM maps have been released including 911 ribosome maps, containing 239 eukaryotic ribosomes, with some of them bound to a ligand (Electron microscopy databank source). Even though there are currently no drugs that were discovered based on cryo-EM structures, one can expect that the growing evidence might guide medicinal chemists towards the synthesis of new bioactive molecules. Some examples include the 2.5 Å resolution structure of the Leishmania donovani ribosome in complex with the antibiotic paromycin [121] and the 3.6 Å structure of the human cytosolic ribosome in complex with cycloheximide, a eukaryote-specific inhibitor of protein biosynthesis [42] (Figure 1F) and also with HHT (Figure 1E) and hygromycin B [25]. All these studies show clear densities for the bound ligands and give insights into their mechanism of action. This knowledge is highly valuable for designing drugs that are more specific to their target and reduces toxicity. Clearly, cryo-EM is potentially playing an important role in structure-guided drug design, in particular for the human ribosome ligand complexes of interest. It is therefore possible for chemists to better understand the interactions between the ribosome and its ligands, and thus manipulate these interactions by chemical modifications.

### Cryo-EM-Based Drug Design of Cycloheximide

Our consortium recently used cryo-EM to visualize with high resolution the binding of the glutarimide compound cycloheximide (CHX) into the 80S ribosome, providing valuable insights into the molecular interactions of the ligand within its ribosomal pocket (Figure 1F) [42].

CHX, originally isolated from *Streptomyces griseus* is a very potent cell toxin in eukaryotes [122]. Numerous synthesis of CHX, as well as of CHX analogues, were performed in the 60s [123]. It has been known for a long time that CHX is interfering with protein synthesis by inhibiting translocation during translation elongation in eukaryotes [124,125]. It, however, has no effect on *E. coli* protein synthesis [126].

**Figure 6:**
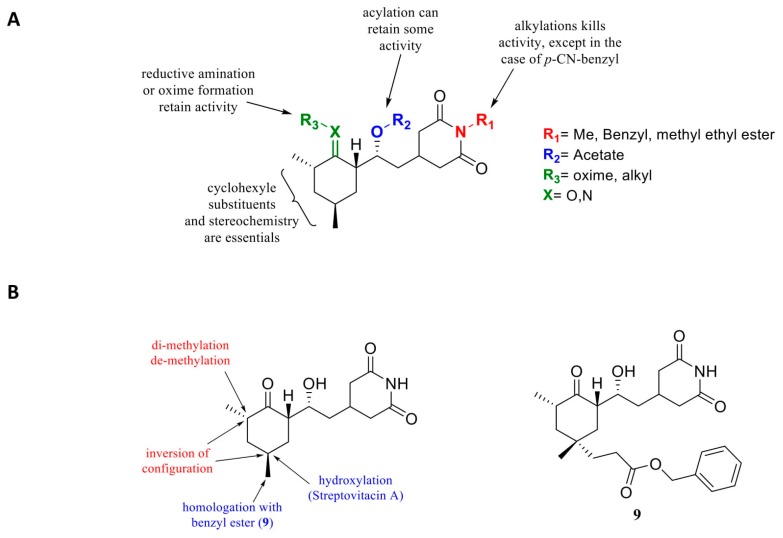
(**A**) Earlier structure activity relationship studies on cycloheximide. (**B**) Cycloheximide derivatives: in red, modifications leading to a loss of activity, in blue, conservation or slight enhancement of activity for compound **5** [132].

The scientific interest for CHX has, however, declined because of its toxicity level, which is too high for the application in humans and because no CHX analogues showed any improvement in activity or toxicity [127,128]. Recently, cycloheximide was derivatized as a fluorescent probe for protein synthesis imaging [129], and also tested as inhibitor of FKBP12 [130] or as an antiviral [131]. These studies have shown that most of the molecular features are essential to maintain its activity (Figure 6). Modification of chiral centers greatly affected activity and should remain identical to the natural compound. These derivatives were chosen empirically, probably because of their easier synthetic accessibility. None of them showed an enhanced bioactivity.

However, it is currently possible to access the structure-guided design of cycloheximide analogues. Consequently, Liau’s group has been able to initiate rational design by modifying CHX [132] from the structure published by [42,132]. Important total synthesis work has been performed providing 11 CHX analogues. They showed that the chirality of the molecules is crucial for CHX activity, also against protein translation (Figure 6A). They also evaluated alterations of the cyclohexanone moiety by incorporating electrophilic groups in order to allow interactions with nearby lysine residues. They noted that incorporation of a butyl chain in the place of a methyl in this part of the molecule did not present a notable decrease in activity but also that adding an electrophilic residue such as benzyl ester improved activity (1 order magnitude) (Figure 6B) on protein synthesis inhibition. However, the compounds did not appear to have better anti-cancer activity in vitro compared to CHX.

This example nevertheless suggests that the structural elucidation of the human cytoplasmic ribosome could give rise to the development of a new class of specific drugs.

We determined that CHX shows a higher activity in leukemic cells compared to normal cells, indicating its potential for anti-cancer drug design [42]. Indeed, the CHX molecule could be extended at the 3,5-dimethyl-2-oxocyclohexyl-moiety towards the position of the E-site tRNA CCA end binding-site, so that the molecule inserts like an E-site tRNA in between G4370 and G4371. Additionally, the space between the CHX and eL42 could further be filled by CHX derivatives to interact with Lys53, Pro54 or Phe56 [42]. In an ongoing project, we synthesized about 70 different CHX derivatives and obtained 5 new molecules displaying 50-100-fold improved anti-leukemic activity compared to parental CHX and a higher inhibitory effect on the ribosome. The characterization of these CHX derivatives is currently being carried out both in vitro and in vivo. Additionally, we are analyzing their toxicity to evaluate potential therapeutic windows.

## 6. Future Perspectives

The key points presented in this review suggest that targeting the ribosome in cancer cells will preferentially affect them, compared to normal cells, as many cancers develop addictions to protein synthesis and to the constitutive activation of the mTORC1 pathway. Moreover, many oncoprotein drivers (MYC, FLT3-ITD, BCR-ABL) or proteins involved in cell survival (MCL-1, XIAP) display a short half-life and consequently appear highly sensitive to ribosome inhibition. Combining ribosome inhibition with either classical chemotherapeutic drugs, targeted therapies or immunotherapies could result in higher lethal effects to boost the efficacy of current treatments.

It has been envisioned that cancer cells might express oncoribosomes that differ in their composition and functions compared to normal ribosomes, and participate in the transformation process (Figure 2). Ribosomes from normal cells are heterogeneous and specialized. In non-transformed ES cells, several RPs: eS7(RPS7), eS25 (RPS25), uL1 (RPL10A), eL38 have been shown to be in substoichiometric abundance, demonstrating the existence of different ribosome subpools [133]. Furthermore, ribosomes with variations in RP composition were found to selectively translate distinct mRNA repertoires. Indeed, ribosomes expressing eS25 favor translation of mRNA associated with organelle organization, cell cycle, and morphogenesis involved in differentiation. However, ribosomes expressing uL1 have a preference for mRNA associated with system development and extracellular matrix organization [133]. Heterogeneity can also come from the existence of paralogs of several RPs that can be expressed in a tissue-specific manner, to compete with, or to regulate the expression of their corresponding RPs [134]. Variations in rRNA, in particular of the 28S species, may also modify ribosome structure as well as the spectrum of its post-translational modifications [13,135]. Cancer transformation is likely to affect, independently or concomitantly, several of these functions, thus converting ribosomes into oncoribosomes. First, some cancers harbor mutated RP genes that predispose to transformation [35]. The oncogenic process can modify the expression of RPs and RP paralogs to change ribosome function, and copy number variations of several RP genes have been shown to be predictive of cancer progression [136]. Oncogenic signaling might trigger different post-translational events on both RPs and rRNA and may also modulate the ribo-interactome, thereby shifting ribosomal properties into a cancer-promoting manner, for instance by altering their cellular localization and render them independent of upstream negative regulatory signals or by favoring the translation of a cancer-specific mRNA repertoire.

The potential of the cryo-EM approach to provide high resolution images of the ribosome with ligands is a powerful guide for drug design strategies aiming at developing more specific and potent inhibitors. Cryo-EM could also help characterize oncoribosomes, and provide structural support for the design of oncoribosome-specific inhibitors, that will spare normal ribosomes and reduce toxicity and side effects.

## Figures and Tables

**Table 1:** Combinations of homoharringtonine with other chemotherapeutic drugs that show potentiating effects.

Cancer Type	Drug	Target	Reference
AML	AraC	anti-metabolite	[113]
AML	aclarubicin	anthracyclin	[107]
AML	decitabine	methylation inhibitor	[109]
AML	abivertinib	BTK inhibitor	[110]
AML	IPI504	HSP90 inhibitor	[111]
AML	etoposide	DNA topoisomerase II	[113]
t(8, 21)AML	oridonin	cKIT, MDR1, MRP-1	[113]
AML	SAHA	HDAC inhibitor	[114]
AML	AsO3	apoptosis induction	[115]
DLBCL	bortezomib	proteasome inhibitor	[116]
BCL-2+ DLBCL	ABT-199	BCL-2	[117]
